# Growth Rate and Biofilm Formation Ability of Clinical and Laboratory-Evolved Colistin-Resistant Strains of *Acinetobacter baumannii*

**DOI:** 10.3389/fmicb.2018.00153

**Published:** 2018-02-12

**Authors:** Zahra Farshadzadeh, Behrouz Taheri, Sara Rahimi, Saeed Shoja, Maryam Pourhajibagher, Mohammad A. Haghighi, Abbas Bahador

**Affiliations:** ^1^Department of Microbiology, School of Medicine, Ahvaz Jundishapur University of Medical Sciences, Ahvaz, Iran; ^2^Department of Medical Laboratory Sciences, School of Paramedicine, Ahvaz Jundishapur University of Medical Sciences, Ahvaz, Iran; ^3^Department of Microbiology, Faculty of Medicine, Bushehr University of Medical Sciences, Bushehr, Iran; ^4^Infectious and Tropical Disease Research Center, Hormozgan Health Institute, Hormozgan University of Medical Sciences, Bandar Abbas, Iran; ^5^Dental Research Center, Dentistry Research Institute, Tehran University of Medical Sciences, Tehran, Iran; ^6^Department of Microbiology, School of Medicine, Tehran University of Medical Sciences, Tehran, Iran

**Keywords:** *Acinetobacter baumannii*, colistin resistance, biofilm formation, growth rate, antimicrobial resistance

## Abstract

Two different mechanisms of resistance to colistin in *Acinetobacter baumannii* have been described. The first involves the total loss of lipopolysaccharide (LPS) due to mutations in the *lpxACD* operon, which is involved in the lipid A biosynthesis pathway. The second entails the addition of ethanolamine to the lipid A of the LPS resulting from mutations in the *PmrAB* two-component system. To evaluate the impact of colistin resistance-associated mutations on antimicrobial resistance and virulence properties, four pairs of clinical and laboratory-evolved colistin-susceptible/colistin-resistant (Col^S^/Col^R^) *A. baumannii* isolates were used. Antimicrobial susceptibility, surface motility, *in vitro* and *in vivo* biofilm-forming capacity, *in vitro* and *in vivo* expression levels of biofilm-associated genes, and *in vitro* growth rate were analyzed in these strains. Growth rate, *in vitro* and *in vivo* biofilm formation ability, as well as expression levels of biofilm-associated gene were reduced in Col^R^ LPS-deficient isolate (the *lpxD* mutant) when compared with its Col^S^ partner, whereas there were not such differences between LPS-modified isolates (the *pmrB* mutants) and their parental isolates. Mutation in *lpxD* was accompanied by a greater reduction in minimum inhibitory concentrations of azithromycin, vancomycin, and rifampin than mutation in *pmr*B. Besides, loss of LPS was associated with a significant reduction in surface motility without any change in expression of type IV pili. Collectively, colistin resistance through loss of LPS causes a more considerable cost in biological features such as growth rate, motility, and biofilm formation capacity relative to LPS modification. Therefore, Col^R^ LPS-modified strains are more likely to spread and transmit from one patient to another in hospital settings, which results in more complex treatment and control.

## Introduction

*Acinetobacter baumannii*, an opportunistic pathogen, is a common causative agent of nosocomial infections around the world. Biofilm-forming ability and antibiotic resistance are considered to be two critical factors for the success of *A. baumannii* as a prevalent nosocomial pathogen (Perez et al., 2007; Durante-Mangoni and Zarrilli, 2011).

In recent years, an increase in the reports of extensively drug-resistant (XDR)-*A. baumannii* strains has been observed, leading to limit treatment options (Lin and Lan, 2014; Durante-Mangoni et al., 2015). In such condition in which therapeutic choices are limited, colistin-based regimens are often the last-resort alternative for treatment of XDR-*A. baumannii* infections (Lin and Lan, 2014). However, alarmingly, global emergence of colistin-resistant (Col^R^) *A. baumannii* strains is increasingly reported to be the consequence of the indiscriminate use of colistin in the hospital settings (Cai et al., 2012; Lin and Lan, 2014). Numerous outbreaks of pan drug-resistant *A. baumannii* have been reported in hospitals in Asia and the Middle East (Méndez et al., 2015).

Colistin is a cationic antimicrobial peptide that affects the lipid A moiety of lipopolysaccharide (LPS) of Gram-negative bacteria, thus disrupting the outer membrane. Two mechanisms of resistance to colistin have been identified in *A. baumannii*. One involves the complete loss of LPS mediated through mutations in the *lpxACD* operon (the *lpxA, lpxC*, or *lpxD* genes). Another mechanism is the modification of the lipid A moiety of LPS resulting from mutations in *pmrAB* (Cai et al., 2012; Nhu et al., 2016). Little is known about the effect of colistin resistance-associated mutations on virulence properties of *A. baumannii*. Considering that colistin resistance-related characterization is a key point to overcome obstacles in the treatment and control of Col^R^ *A. baumannii* infection, understanding the impact of colistin resistance on pathobiological features is critical. There are a number of contradictory reports on changes in several properties such as fitness, antibiotic resistance, and virulence accompanying development of colistin resistance in *A. baumannii* (Fernández-Reyes et al., 2009; Dafopoulou et al., 2015; García-Quintanilla et al., 2015), whereas the biofilm formation capacity, despite its important role in the development of antibiotic resistance and pathogenesis of *A. baumannii*, has been poorly investigated. In these studies, analyzing biofilm-forming ability was restricted to a colorimetric measurement of biofilm mass in relation to colistin resistance (Beceiro et al., 2014; Durante-Mangoni et al., 2015). However, precise examination of biofilm formation capacity needs assessment of expression of biofilm-associated genes from which *csuA/BABCDE, ompA, bfmR/S, pgaABC, Bap*, and *abaI* have been extensively reported to be present in all *A. baumannii* strains (Choi et al., 2009; Gaddy and Actis, 2009). Besides, to our knowledge, no studies have compared the surface motility between Col^R^ isolates and their Col^S^ parental isolates. Accordingly, contradictory reports by previous studies warrant further studies to evaluate characteristics by which colistin resistance acquisition can be followed in Col^R^ *A. baumannii* and especially to analyze surface motility, biofilm mass, and expression of biofilm-associated genes. The present study aims to investigate four pairs of clinical and laboratory-evolved Col^R^/Col^S^ *A. baumannii* isolates to investigate the effect of colistin resistance-associated mutations on the growth rate, antimicrobial resistance, surface motility, *in vitro* and *in vivo* biofilm formation capacity, and expression of biofilm-associated genes.

## Materials and Methods

### Bacterial Isolate Description and Identification

The eight *A. baumannii* isolates used in this study included two pairs of Col^S^/Col^R^ clinical isolates and two pairs of laboratory mutants (**Table 1**). All isolates were initially confirmed as members of the *A. baumannii* complex using the API 20NE Kit (bioMérieux, Marcy-l’Etoile, France). The species level identification of *A. baumannii* was performed by *bla*_OXA-51_-like gene tracking and a multiplex polymerase chain reaction (PCR) method using *gyrB*-directed primers (Higgins et al., 2010).

**Table 1:** Characteristics of *Acinetobacter baumannii* isolates in this study.

						MLVA profiles									
Isolates	Date of isolation	Specimen	Colistin MIC (μg/ml)/status of susceptibility	Sequence group (SG)	CC/ST^d^	MLVA-AB- 3530	MLVA-AB- 3002	MLVA-AB- 2240	MLVA-AB- 1988	MLVA-AB- 0826	MLVA-AB- 0845	MLVA-AB- 2396	MLVA-AB- 3468	Nucleotide substitutions	Amino acid substitutions
Ab1	25 April 2013	CSF^a^	0.25/S^b^	SG2	227/405	11	7	4	9	17	9	N^e^	32	Wilde type	Wild type
Ab2	30 April 2013	CSF	256/R^c^	SG2	227/405	11	7	4	9	17	9	N	32	C697T mutation in *pmr*B	P233S
Ab321	27 December 2015	Burn wound	0.5/S	SG7	92/75	8	6	4	5	9	13	54	15	Wilde type	Wild type
Ab328	3 January 2016	Burn wound	128/R	SG7	92/75	8	6	4	5	9	13	54	15	C697T mutation in *pmr*B	P233S
Ab12	3 October 2015	Burn wound	0.5/S	SG9	92/118	10	7	4	9	52	13	66	30	Wild type	Wild type
Ab12R	8 October 2015	Laboratory mutant	256/R	SG9	92/118	10	7	4	9	52	13	66	30	G739T mutation in *lpx*D	Nonsense mutation
Ab99	7 December 2015	Burn wound	0.5/S	SG9	92/118	10	7	4	9	52	13	66	30	Wild type	Wild type
Ab99R	15 December 2015	Laboratory mutant	128/R	SG9	92/118	10	7	4	9	52	13	66	30	C695T mutation in *pmr*B	T232I

*^*a*^CSF, cerebral spinal fluid; ^*b*^S, susceptible; ^*c*^R, resistant; ^*d*^CC, clonal complex; ST, sequence type; ^*e*^N, not amplified.*

### Colistin Resistance Determination

For phenotypic determination of colistin resistance, the minimum inhibitory concentrations (MICs) to colistin were initially determined by MIC test strip (Liofilchem, Italy), and finally confirmed by the glass tube dilution method (Clinical and Laboratory Standards Institute [CLSI], 2015a). For genotypic determination of colistin resistance, PCR sequencing to detect the genes *pmrA, pmrB, pmrC, lpxA, lpxC*, and *lpxD* were carried out as described previously (Pournaras et al., 2014). Basic Local Alignment Search Tool (BLAST) software was used to determine the amino acid sequences from the nucleotide sequences obtained by PCR. Then, the BLAST output amino acid sequences of all test genes were compared with the deduced amino acid sequences available in GenBank.

### Preparation of Col^R^ Laboratory Mutants of *A. baumannii*

In order to examine differences in virulence features accompanying colistin resistance between clinical and laboratory-mutant isolates, *lpx* and *pmr* mutants were developed in the laboratory for this study. In the previous study, we had identified the clonal complex (CC) 92/sequence type (ST) 118 as the most prevalent clone. Two independent isolates (Ab12 and Ab99) belonging to CC92/ST118 were selected for generation of laboratory mutant (Farshadzadeh et al., 2015). The Col^R^ mutant (Ab12R), with an *lpx* mutation, was selected by direct plating of its Col^S^ clinical parent (Ab12) onto Mueller-Hinton agar (Merck, Germany) containing 10 μg/ml of colistin sulfate (Sigma). The Col^R^ colonies were then identified following a single round of selection (Moffatt et al., 2010, 2011). The Col^R^ mutant Ab99R with a *pmr* mutation was isolated from the susceptible clinical isolate Ab99 by serial passage in LB broth (Merck, Germany) with increasing concentrations of colistin (1, 2, 4, and 8 μg/ml) (Beceiro et al., 2011). The Col^R^ laboratory mutants of *A. baumannii* were confirmed as described in the previous section.

### Antimicrobial Susceptibility Testing

Susceptibility to piperacillin, ticarcillin, imipenem, meropenem, cefotaxime, ceftazidime, cefepime, ceftriaxone, piperacillin-tazobactam, ampicillin–sulbactam, gentamicin, amikacin, tobramycin, tetracycline, minocycline, doxycycline, ciprofloxacin, rifampin, tigecycline, vancomycin, azithromycin, and colistin was determined by the broth microdilution method according to the CLSI 2015 guideline (Clinical, and Laboratory Standards Institute [CLSI], 2015b). Since there are no available breakpoints for *A. baumannii* strains for tigecycline, rifampin, vancomycin, and azithromycin in the CLSI guidelines, criteria for interpretation of MIC values of tigecycline was determined according to the European Committee on Antimicrobial Susceptibility Testing (EUCAST) (European Committee on Antimicrobial Susceptibility Testing [EUCAST], 2014) for members of the *Enterobacteriaceae.* CLSI criteria for *Staphylococcus* spp. was applied to the other aforementioned antibiotics. *Escherichia coli* (ATCC 25922) and *Pseudomonas aeruginosa* (ATCC 27853) were used as quality control organisms to ensure accuracy of the antimicrobial susceptibility assays, and *E. coli* (ATCC 35218) was used as a quality control organism for β-lactam/β-lactamase inhibitor combinations of antibiotics (piperacillin–tazobactam, ampicillin–sulbactam). All antibiotic powders were purchased from Sigma–Aldrich Company, Germany. The *A. baumannii* phenotype is defined as MDR and XDR according to the international expert proposal for interim standards guidelines (Magiorakos et al., 2012).

### Molecular Typing

Each pair of the Col^S^/Col^R^ clinical isolate (Ab1/Ab2 and Ab321/Ab328) was isolated from a single hospitalized patient. In our study in order to confirm that Col^R^ isolate originates from Col^S^ isolate three typing method was performed, as having identical molecular typing pattern was defined as a single clone. To better understanding genetic relatedness of Ab12 and Ab99, molecular typing of mentioned clinical isolates was also performed in this study. The sequence group (SG) or international clone (IC) type of all Col^S^/Col^R^ strains that were consecutively isolated from the hospitalized patients were identified by using two complementary multiplex PCR sets as previously described (Turton et al., 2007). Col^S^/Col^R^ clinical isolates were grouped in SG2 (IC I), SG1(IC II), SG3 (IC III), or variant (V) clonal type. The multi-locus variable number tandem repeat analysis (MLVA) typing of all isolates were performed by using the MLVA-8 scheme method developed by Pourcel et al. (2011). The MLVA-8 scheme profiles in each isolate were identified by the number of repeats estimated at each VNTR locus according to the predefined order of the previous study (Pourcel et al., 2011). Also each pairs were further investigated with the multi-locus sequence typing (MLST) scheme described by Bartual et al. (2005). By using the *Acinetobacter* MLST database^1^ the allelic profiles of the seven housekeeping genes and STs of the strains were recognized.

### Detection of Genes Involved in Biofilm Formation

Genomic DNA was extracted from each isolate separately using a Bacterial DNA Extraction Kit (GeneAll, Seoul, South Korea). The primers for one set of multiplex PCR reactions were designed to amplify *csuE, bfmR, bfmS, abaI, ompA, bap*, and *pgaA* (Supplementary Table S1). The detection of recently investigated genes involved in biofilm formation through attachment and surface motility including the type I and IV pili representative genes, A1S_1510 and *PilT*, respectively, was separately performed. All pairs of primers were designed using published DNA sequences of *A. baumannii* genes available from the National Center for Biotechnology Information (NCBI) using Primer3web software (version 4.0.0^2^). PCR primers were synthesized by Macrogen (Seoul, South Korea). To set the annealing temperature of each pair of primers, PCR was performed for each pair of primers in a total volume of 25 μl with the following in a reaction tube: 2 μl of DNA template, 10 μl of Amplicon Master Mix, 1 μl of each forward and reverse primer (2.5 pmol), and 11 μl of distilled water. The annealing temperature of all primers was 55°C.

### *In Vitro* Bacterial Biofilm Assay

#### Microplate Biofilm Assay

The biofilm formation assay was performed in triplicate using crystal violet (CV) staining according to Zhang et al. (2016). Briefly, each *A. baumannii* isolate was cultured on brain heart infusion (BHI) agar (Merck, Germany) overnight, and a colony of each isolate was suspended in LB broth (Merck, Germany) and incubated at 37°C for 4 h. Bacterial suspensions were adjusted to an optical density of 0.1 at 600 nm and 200 μl was added to each well in flat-bottomed 96-well sterile cell culture plates (SPL Life Sciences, Gyeonggi-do, South Korea). Following a 48 h incubation period at 37°C, non-adherent bacteria were removed by washing three times with distilled water. The remaining adherent bacteria were stained by adding 200 μl of 0.1% CV to each well. Following incubation of the plates at room temperature for 30 min, the plates were washed three times with distilled water and dried. Then, 100 μl of 95% ethyl alcohol was added to each well and the absorbance of each well was measured at 570 nm. The reference strain ATCC DH5α was used as a negative control (NC). The mean NC absorbance was used to assign scores for biofilm formation of each isolate, as follows: Negative (N) = [A] ≤ [NC], weakly positive (W) = [NC] < [A] ≤ [2NC], moderately positive (M) = [2NC] < [A] ≤ [4NC], and strongly positive (S) = [A] > [4NC].

#### Catheter-Associated Biofilm

To assess the ability of the Col^S^ and Col^R^ *A. baumannii* strains to adhere to a catheter, a 14-gage silicone urinary tract catheter was aseptically cut into 1 cm segments, each of which was placed into a well of a sterile, 12-well flat-bottomed tissue culture plate (SPL Life Sciences, Gyeonggi-do, South Korea) containing 2 ml of bacterial cell suspension with a final concentration of 10^6^ colony forming units (CFU)/ml in exponential growth phase. All segments were incubated for 2 h at 37°C. After the incubation period, samples were washed three times with 15 ml 0.01 M PBS to remove the non-adherent bacteria. Then each catheter section was placed in a 50 ml sterile centrifuge tube containing 10 ml of PBS, and sonicated (Branson 1510 RMT, 70W, 42 KHz, Danbury, CT, United States) for 10 min. For each sample, the bacterial concentration (CFU/ml) was calculated using the Miles et al. (1938) method.

### *In Vivo* Bacterial Biofilm Assay

All animal experiments were carried out in accordance with protocols approved by the Animal Ethics Committee of Tehran University of Medical Sciences (Application No. 92-03-30-23186). In order to investigate the ability of bacteria to form biofilm *in vivo* and to evaluate the *in vivo* effect of colistin resistance on expression levels of genes involved in biofilm formation, as described previously (Kadurugamuwa et al., 2003) 27 female *BALB/c* mice (22–24 g) were divided into nine groups, with three mice in each group and one mouse per cage. All mice were anesthetized with ketamine (10%) at 100 mg/kg and xylazine (2%) at 5 mg/kg. A 2 × 2 cm area of dorsal skin was shaved, and exposed skin was cleansed with iodine and 70% ethanol. A subcutaneous pocket was created with an 8–10 mm incision on the back of each mouse. Then an intravenous catheter (1 cm segment) was implanted into the incision. The skin was closed with surgical staples. In the experimental groups, infection was induced by the injection of 300 μl of bacterial suspension (3 × 10^6^ CFU/ml) under the skin pocket (Kadurugamuwa et al., 2003). The three mice in the control group were injected with PBS, four experimental groups were injected with Col^S^ (Ab1, Ab321, Ab12, and Ab99), and the other four experimental groups were injected with Col^R^ (Ab2, Ab328, Ab12R, and Ab99R) *A. baumannii* isolates. After 7 days, the animals were sacrificed and the catheters were removed. Any biofilm that formed on each catheter was quantified by two different spectrophotometric methods. The 2, 3-bis-(2-methoxy-4-nitro-5-sulfophenyl)-2H-tetrazolium-5-carboxanilide (XTT) assay was measured at 492 nm to determine the biofilm metabolic activity, and the CV assay was measured at 570 nm to determine the total biomass (Cobrado et al., 2013).

### Real-Time Quantification Polymerase Chain Reaction (qRT-PCR) for Biofilm Gene Expression

The gene expression level of the *abaI, ompA, bfmR, bfmS, csuE, bap, pgaA, pilT*, and A1S_1510 genes was examined in the Col^R^ and Col^S^ *A. baumannii* isolates grown under three different conditions: planktonic, *in vitro* biofilm, and *in vivo* biofilm. The RNA extraction (High Pure RNA Isolation Kit, Roche, Germany) and cDNA synthesis (cDNA Synthesis Kit, Thermo Scientific, United States) for each isolate were performed according to the manufacturers’ instructions. The levels of expression of the genes involved in biofilm formation were measured in triplicate using SYBR Premix Ex Taq II (Takara Bio, Inc., Japan). Using the ABI Step One^TM^ System (Applied Biosystems, San Francisco, CA, United States), quantitative real-time (qRT)-PCR was performed in a 25-μl total reaction volume containing cDNA and specific primers. RT-PCR was carried out with the following cycle profile: 1 cycle at 95°C for 30 s, followed by 40 cycles at 95°C for 5 s, 60°C for 10 s, and 72°C for 20 s. The housekeeping gene 16S rRNA was used as an internal control to normalize the levels of each gene transcript. The fold changes of the target gene expression levels were calculated by the 2^-ΔΔC_T_^ method. Differentially expressions of genes were analyzed with the use of the criteria threshold of twofold change (Rajeevan et al., 2001; Etienne et al., 2004). Then these differences were assessed by Student’s *t*-test for consideration as statistically significant. A *P*-value of ≤0.05 was considered as a significant level. The primer sequences used in the qRT-PCR are described in Supplementary Table S1.

### Motility Assay

The surface motility characteristics were investigated on motility assay plates prepared with 5 g/l tryptone, 2.5 g/l NaCl, and the addition of 5 g agarose/l (0.3%) (Merck, Germany). After overnight culture of the strains in LB agar plate, a single colony was inoculated into 5 ml of LB broth and incubated to stationary phase at 37°C with shaking at 180 rpm. After incubation, 2 μl of the bacterial LB broth cultures with an OD_600_ of 2 was placed on the surface of the motility plates and incubated at 37°C for 18 h prior to performing the motility assay. Five independent repeats were done for each experiment, and data were calculated as the mean ± standard deviation (SD) (Harding et al., 2013).

### Growth Curve

The *in vitro* growth rate was assessed for four pairs of isolates by diluting 1 × 10^6^ CFU/ml of each isolate in exponential growth phase in LB broth, and incubating at 37°C with constant shaking at 180 rpm. *A. baumannii* strain ATCC 19606 was used as a control strain. At 12, 24, 36, and 48 h, a 10-fold serial dilution of each culture was spread on an antibiotic-free LB agar plate, and the number of CFUs was counted after 24 h incubation at 37°C. For each strain, three independent experiments were carried out at each time point, and the growths of the strains were compared using statistical analysis as described below (Pournaras et al., 2014).

### Statistical Analysis

Statistical analyses were carried out by one-way analysis of variance (ANOVA) and Student’s *t*-test to evaluate the reduction in growth rate, ability to form biofilm after confirmation of resistance to colistin, and the differences between the relative quantities of the gene expressions in Col^S^ and Col^R^ *A. baumannii* strains were determined. The effect of the mutations causing resistance to colistin on the reduction of MIC to antimicrobial agents in *A. baumannii* strains was evaluated by the Mann–Whitney *U*-test. All analyses were performed using IBM SPSS version 22. *P*-values ≤ 0.05 in all experiments were considered statistically significant.

## Results

### Bacterial Isolates

#### First Col^S^/Col^R^ *A. baumannii* Pair (Ab1/Ab2)

Ab1/Ab2 pair was isolated from a 20-year-old hospitalized man with an abdominal gunshot trauma. The patient underwent laparotomy three times. There was a debrided ulcer on his back with a leakage of cerebral spinal fluid (CSF). On the day of admission a CSF sample was taken and cultured. After growth, colistin-susceptible *A. baumannii* (Ab1) was recovered. Therefore, colistin therapy in combination with rifampin (intravenous and intrathecal) was initiated after 4 days. On the fifth day of colistin administration, another CSF sample was taken and cultured. After incubation for 18 h, Col^R^ *A. baumannii* (Ab2) was isolated.

On the 13th day of treatment with colistin, a CSF sample was taken, analyzed, and cultured. Although CSF analysis showed meningitis pattern, the culture was negative for *A. baumannii.* Colistin treatment was continued until the last day of his life (26 days of hospitalization). These isolates were previously described (Moosavian et al., 2014).

#### Second Col^S^/Col^R^ *A. baumannii* (Ab321/Ab328)

A 28-year-old female patient, who had attempted suicide by burning, was admitted to burn unit with a major thermal injury. She had sustained approximately 73% total body surface area (TBSA). This injury caused third grade burns in arms, upper and lower limbs, thorax, and abdomen. Systemic dehydration was compensated with fluid resuscitation and supportive care and first debridement also was done. On the fifth day, she had developed high grade fever and sudden tachycardia. Sepsis progressed to severe form and septic shock. Colistin-susceptible *A. baumannii* (Ab321) was recovered from blood culture. Ampicillin–sulbactam, meropenem, and colistin were administered in different combination. After 4 days, Col^R^ *A. baumannii* (Ab328) was recovered from blood culture. Treatment with tigecycline and colistin combination was started. After 5 days, the general condition of patient was better and discharged 9 weeks later. Both Col^R^ clinical isolates, which were isolated from two different patients after colistin therapy, harbored the *pmrB* mutation.

### *A. baumannii* Strain Sequence Characteristics from PCR-Sequencing

Nucleotide sequencing of the genes involved in colistin resistance showed that there were mutations in *pmrB* and *lpxD* genes, whereas no mutations in the *pmrA, pmrC, lpxA*, and *lpxC* genes were found. Comparison of nucleotide sequences of the *pmrB* gene between Col^R^ and Col^S^ isolates indicated that Col^R^ isolates, Ab2 and Ab328, have a C697T single-nucleotide mutation (P233S). Another point mutation in the *pmrB* gene which causes a C-to-T transition (T232I) at nucleotide 695 was identified in isolate Ab99R, similar to reported mutations by previous studies (Lesho et al., 2013; Pournaras et al., 2014). Similarly, evaluation of nucleotide sequences of the *lpxD* gene in Col^R^ and Col^S^ isolates showed that Col^R^ isolate, Ab12R, has one mutation (G739T), which has previously been described as a premature stop (nonsense) codon mutation in LPS-deficient isolates (García-Quintanilla et al., 2015). Phenotypic and genotypic characteristics of all isolates are presented in **Table 1**.

### Antimicrobial Susceptibility Testing

The results of antimicrobial susceptibility testing are shown in Supplementary Table S2 and MICs to colistin are shown in **Table 1**. All isolates in this study were found to be resistant to carbapenems (imipenem and meropenem). All Col^S^ isolates (Ab12, Ab99, Ab1, and Ab321) were resistant to all clinically relevant antibiotics and exhibited a XDR phenotype. Similarly, three Col^R^ isolates including one laboratory mutant, Ab99R, and two clinical isolates, Ab2 and Ab328, were found to have a XDR phenotype, while laboratory Ab12R mutant was shown to have acquired a MDR phenotype along with colistin resistance.

As shown in Supplementary Table S2, the MIC values of rifampin (16-fold), azithromycin (128-fold), and vancomycin (256-fold) in laboratory-derived Col^R^ strain (LPS-deficient Col^R^ strain), Ab12R, were significantly lower than those in its parent, whereas the values for amikacin, gentamicin, tobramycin, imipenem, meropenem, ticarcillin, and cefepime were moderately different; for piperacillin, piperacillin–tazobactam, ampicillin–sulbactam, cefotaxime, and ceftazidime were minimally various; and for ceftriaxone, tetracycline, minocycline, and doxycycline were similar. Interestingly, the MIC values for tigecycline and ciprofloxacin LPS-deficient Col^R^ strain were higher relative to its partner. However, such significant differences were not found between three LPS-modified Col^R^ isolates (the *pmrB* mutants) and their counterparts.

### Molecular Typing

As shown in **Table 1**, each Col^S^ and Col^R^ clinical isolate within a pair had identical site groups (SGs), MLVA profiles, and STs. Ab1 and its pair, Ab2, belonged to SG2, whereas two pairs of isolates including Ab12/Ab12R, Ab99/Ab99R were assigned SG9 and Ab321/Ab328 was grouped as SG7, as described previously (Karah et al., 2012). Isolates that were assigned to the SG9 V type, Ab12 and Ab99, belonged to the previously known ST118. ST118 belongs to CC92. As Ab12 and Ab99 have identical SG, allelic MLVA profile and ST, this indicates that these isolates have the same ancestral lineage. On the other hand, Ab1/Ab2 and Ab321/Ab328 belong to ST405 (CC227) and ST75 (CC92), respectively. The MLVA profiles of all isolates are shown in **Table 1**.

### *In Vitro* and *in Vivo* Biofilm Assay

The biofilm formation abilities of the Col^S^ isolates and their Col^R^ counterparts are compared in Supplementary Table S3. The microplate biofilm assay, catheter-adhesion assay, and *in vivo* analysis of the biofilms revealed that all Col^S^ isolates have a strong capacity to form bacterial adhesion and strong biofilms on the catheter (Supplementary Table S3). Like their counterparts, LPS-modified Col^R^ isolates, Ab2, Ab328, and Ab99R, developed strong biofilm (*P* > 0.05), whereas the ability of LPS-deficient Col^R^ isolate, Ab12R, to produce biofilm was significantly lower than its partner, Ab12 (*P* < 0.05). There was a one log reduction in the level of *in vitro* adhesion for Ab12R versus its parent Ab12 (*P* = 0.001). No significant reduction in biofilm formation among the other Col^R^ isolates was observed (*P* > 0.05).

### Gene Expression Profiles of Exponential-Phase Planktonic Cells, *in Vitro*, and *in Vivo* Biofilm Cells

After confirming the presence of the *ompA, abaI, bap, bfmR, bfmS, csuE, pgaA*, and A1S_1510 genes in all isolates using PCR, the expression levels of these genes were compared between Col^R^ isolates and their Col^S^ partners in exponential-planktonic, *in vitro* biofilm, and *in vivo* biofilm phases. As shown in **Table 2**, statistical comparison of the relative expressions of the genes revealed that the LPS-deficient Col^R^ isolate, Ab12R, was expressed in these genes under *in vitro* and *in vivo* biofilm conditions at a lower level relative to its Col^S^ parent, while these genes were not differentially expressed between the LPS-modified Col^R^ isolates, Ab99R, Ab2, and Ab328, and their Col^S^ partners. Besides, no differences were seen in the relative expressions of the genes between Col^S^ isolates and their Col^R^ parents in the planktonic phase. The *pilT* gene involved in the assembly of type IV pili did not exhibit different expression between all Col^S^ isolates and their Col^R^ parents under planktonic, *in vitro*, and *in vivo* biofilm conditions.

**Table 2:** Expression levels of genes in planktonic cells, *in vitro* and *in vivo* biofilm modes between colistin-susceptible when compared with colistin resistance strains as measured by qRT-PCR.

Phases	Genes	Ab12/Ab12R fold change	*P*-value	Ab99/Ab99R fold change	*P*-value	Ab1/Ab2 fold change	*P*-value	Ab321/Ab328 fold change	*P*-value
Planktonic phase	*abaI*	0.72	0.08	0.73	0.07	1.02	0.12	1.03	0.11
	*bfmR*	1.04	0.11	1.06	0.09	1.10	0.16	1.05	0.12
	*bfmS*	1.07	0.12	1.10	0.17	1.02	0.17	1.16	0.12
	*csuE*	1.20	0.16	1.04	0.18	1.00	0.13	0.67	0.08
	*pgaA*	1.02	0.12	1.05	0.13	0.93	0.11	1.19	0.17
	*ompA*	0.93	0.11	1.07	0.14	1.03	0.12	1.11	0.14
	*bap*	1.01	0.13	1.05	0.16	1.31	0.14	1.01	0.11
	*A1S_1510*	1.20	0.18	1.22	0.21	1.61	0.19	1.08	0.13
	*pilT*	0.88	0.11	0.99	0.11	0.77	0.11	0.93	0.12
*In vitro* biofilm mode	*abaI*	2.83	0.04	1.17	0.11	1.23	0.18	1.10	0.12
	*bfmR*	2.61	0.02	1.04	0.17	1.13	0.12	0.98	0.09
	*bfmS*	2.73	0.01	1.06	0.14	1.17	0.16	1.27	0.17
	*csuE*	3.36	0.02	0.97	0.12	1.05	0.14	0.92	0.13
	*pgaA*	2.32	0.04	1.13	0.18	0.87	0.11	1.06	0.12
	*ompA*	2.38	0.04	1.31	0.21	1.53	0.16	1.42	0.18
	*bap*	2.12	0.03	1.18	0.19	1.33	0.14	1.09	0.09
	*A1S_1510*	2.29	0.03	1.21	0.20	1.48	0.17	1.12	0.15
	*pilT*	1.21	0.13	1.09	0.16	0.89	0.10	1.06	0.11
*In vivo* biofilm mode	*abaI*	3.11	0.02	1.02	0.17	1.39	0.15	1.28	0.16
	*bfmR*	5.76	0.001	1.14	0.17	1.01	0.12	1.03	0.11
	*bfmS*	2.87	0.03	1.04	0.14	1.05	0.13	0.97	0.09
	*csuE*	3.62	0.01	1.05	0.13	1.03	0.11	1.16	0.18
	*PgaA*	2.48	0.04	1.04	0.11	1.04	0.12	0.94	0.11
	*ompA*	2.55	0.03	1.09	0.12	1.39	0.16	1.38	0.17
	*bap*	2.33	0.04	1.46	0.25	1.23	0.15	1.05	0.11
	*A1S_1510*	2.46	0.04	0.97	0.11	1.57	0.17	1.18	0.18
	*pilT*	1.37	0.13	0.84	0.12	0.82	0.09	0.93	0.09

### Motility Assay

We then examined the motility of all isolates on motility plates. Calculating the mean diameter of colonies indicated that there were no significant difference in colony sizes between three LPS-modified Col^R^ isolates and their Col^S^ parental isolates (*P* > 0.05), whereas the LPS-deficient Col^R^ isolate formed a significantly smaller colony compared with its Col^S^ counterpart (*P* = 0.035) (Supplementary Table S3).

### Growth Analysis

**FIGURE 1:**
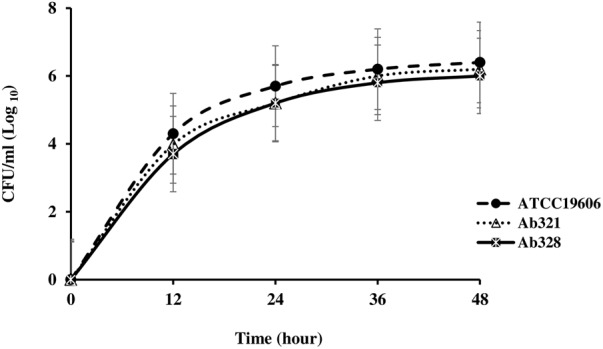
Growth curves of reference strain ATCC 19606 and colistin-susceptible isolate: Ab321 and respective colistin-resistant isolate: Ab328. The growth rates between Col^R^ isolate, Ab328, and their respective Col^S^ isolate, Ab321, were not significantly different at any time points (*P* > 0.05).

**FIGURE 2:**
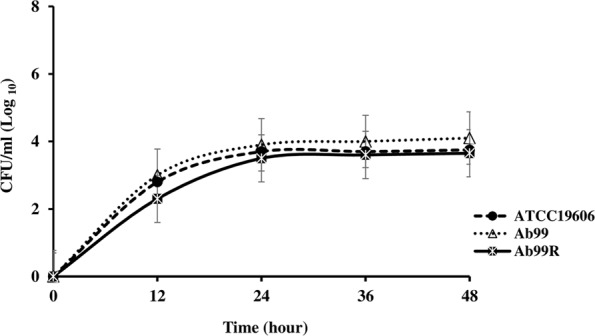
Growth curves of reference strain ATCC 19606 and colistin-susceptible isolate: Ab99 and respective colistin-resistant isolate: Ab99R. The growth rates between Col^R^ isolate, Ab99R, and their respective Col^S^ isolate, Ab99, were not significantly different at any time points (*P* > 0.05).

**FIGURE 3:**
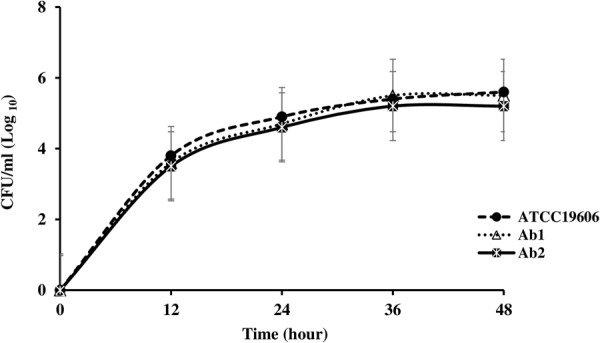
Growth curves of reference strain ATCC 19606 and colistin-susceptible isolate: Ab1 and respective colistin-resistant isolate: Ab2. The growth rates between Col^R^ isolate, Ab1, and their respective Col^S^ isolate, Ab2, were not significantly different at any time points (*P* > 0.05).

**FIGURE 4:**
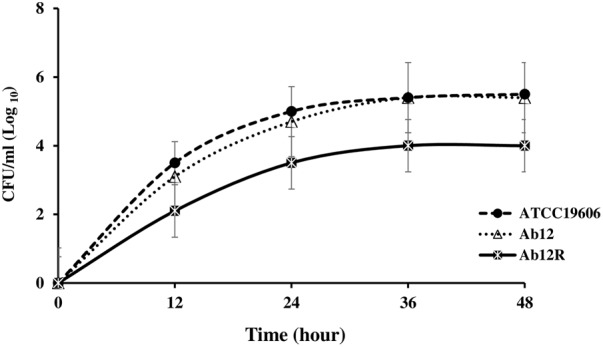
Growth curves of reference strain ATCC 19606 and colistin-susceptible isolate: Ab12 and respective colistin-resistant isolate: Ab12R. There was a significant (*P* < 0.05) difference in cell count at all times points between susceptible isolate Ab12 and its resistant partner Ab12R.

The growth curves of each of the Col^S^/Col^R^ paired isolates and the ATCC 19606 control strain are shown in **Figures 1–4**. The growth of the Col^R^ laboratory isolate Ab12R was remarkably slower than that of the Col^S^ parent Ab12. There were significant differences (*P* < 0.05) in cell count at all time points between Ab12 and Ab12R, whereas in all other Col^S^/Col^R^ pairs (Ab99/Ab99R, Ab1/Ab2, and Ab321/Ab328), the growth rates between the Col^R^ isolates and the parental Col^S^ isolates were not significantly different at any time points (*P* > 0.05). In addition, the colony sizes of the Ab12R isolate in the antibiotic-free LB agar plate were noticeably smaller than those of Ab12. There were no colony size differences between other Col^R^ isolates (Ab99R, Ab2, and Ab328) and their partners (Ab99, Ab1, and Ab321).

## Discussion

The clinical significance of Col^R^ *A. baumannii* strains has recently been highlighted with reports on the emergence of colistin resistance after colistin treatment of an infection caused by a multidrug-resistant strain. There are a number of reports on several biological characteristics of these Col^R^ strains, but biofilm formation capacity, despite being of special importance in the virulence of *A. baumannii*, has only scarcely been studied for its phenotypic characteristics. In the present study, we evaluated the biofilm formation capacity, growth rate, and antibiotic resistance of two laboratory mutant Col^R^ *A. baumannii* strains.

In the present study, the LPS-deficient Col^R^ isolate, Ab12R, was dramatically more susceptible to vancomycin, azithromycin, and rifampin when compared with its Col^S^ partner, while three LPS-modified Col^R^ isolates, Ab99R, Ab2, and Ab328, did not have an increase in sensitivity to these antibiotics relative to their Col^S^ partners. Therefore, the resistance to colistin via loss of LPS has a greater effect on increasing antibiotic-susceptibility relative to modification of LPS, demonstrating that changes in permeability of outer membrane lead to increasing susceptibility to some antibiotics. This finding suggests that the mentioned antibiotics could be helpful in the development of antimicrobial synergisms to treat infections caused by LPS-deficient Col^R^ *A. baumannii*. These results are in agreement with previous studies that showed that LPS plays a critical role in providing a barrier to hydrophobic antibiotics and compounds (García-Quintanilla et al., 2015).

The acquisition of colistin resistance in LPS-deficient Col^R^ isolate was associated with a significant decrease in growth rate, whereas the growth rates of three LPS-modified Col^R^ isolates were similar to their Col^S^ parents. In agreement with the present study, the *pmrB* C697T mutation in previous studies did not significantly associate with reduced growth rate (Beceiro et al., 2014; Durante-Mangoni et al., 2015). Inconsistently, in another study by Yoon et al. (2013), *pmrA* or *pmrB* mutations in 14 clinical Col^R^ *A. baumannii* isolates were shown to be related to a reduction in both fitness and virulence.

The evaluation of several pathobiological properties including surface attachment, surface motility, and *in vitro* and *in vivo* biofilm formation capacity revealed that the acquisition of colistin resistance in the LPS-deficient Col^R^ strain was associated with a remarkable decrease in these pathobiological properties. Interestingly, biofilm-forming ability in three LPS-modified Col^R^ isolates was not significantly different from their Col^S^ counterparts. It is important to note that the precise examination of biofilm formation capacity needs assessment of expression of biofilm-associated genes, because altering biofilm formation capacity could be related to change in growth rate. However, previous researches investigating the impact of colistin resistance on biofilm-forming ability were restricted to a colorimetric measurement of biofilm mass (Beceiro et al., 2014; Dafopoulou et al., 2015; Durante-Mangoni et al., 2015). One of these studies investigated biofilm formation ability only by photometric assay and revealed that the acquisition of colistin resistance via the *pmrB* C697T is associated with a decrease in both biofilm formation capacity and growth rate (Dafopoulou et al., 2015). However, it is possible that decreased capacity of biofilm formation was due to reducing growth rate. In the present study, in order to confirm that decreasing biofilm-forming ability was due to reducing ability to develop biofilm, but not increasing growth rate, the relative expressions of biofilm-associated genes were assessed. Our data revealed that the expression levels of the *bap, bfmR, bfmS, abaI, csuE, pgaA, ompA*, and A1S_1510 genes in LPS-deficient Col^R^ *A. baumannii* strain, Ab12R, were higher than those in its partner under *in vitro* and *in vivo* biofilm conditions. The *AbaI* protein mediated production of the primary quorum sensing (QS) signal *N*-acyl-homoserinelacton (AHL). Niu et al. (2008) demonstrated that the ability to form biofilm in the isogenic *abaI*::Km mutant was lower than wild type. These results indicate that the expression of the *abaI* gene is linked to the formation of biofilm. The expression of *AbaI* as an autoinducer is elevated in response to fluctuations in cell population density and biofilm formation (Niu et al., 2008; Roca et al., 2012). The *CsuA/BABCDE* chaperon-usher (CU) assembly system assembling type I pili (CU pili) in *A. baumannii* plays an important role in surface attachment and biofilm formation. The *csu* operon is regulated by a two-component system, *bfmR*/*bfmS* (Tomaras et al., 2008; Cerqueira and Peleg, 2011). Second type I pili cluster reported to be involved in surface adherence and biofilm formation is A1S_1510-1507 cluster (Rumbo-Feal et al., 2017). Analysis of the expression of the representative gene of each type I pili cluster revealed a reduction in their expression following development of colistin resistance through loss of LPS, but not through modification of LPS. *A. baumannii* strains produce two surface-associated proteins, *Bap* and *OmpA*, which are important for the development of mature biofilm on abiotic and biotic surfaces (Rumbo-Feal et al., 2017). Biofilm formation capacity of *A. baumannii* also depends on glycosylation system to produce poly-β-1, 6-*N*-acetylglucosamine (PNAG). This major component of the biofilm exopolysaccharidic matrix is encoded by the *pgaABCD* cluster (Choi et al., 2009). However, Rebekah et al. demonstrated that increasing expression of *pgaABCD* in exponential-planktonic phase cells of LPS-deficient *A. baumannii* ATCC 19606 do not lead to increasing levels of biofilm (Henry et al., 2012). A possible explanation is that the glycosylation system *pgaABCD* may be involved not only in biofilm formation, but also in the other processes such as glycosylation of surface-exposed proteins and lipoproteins that could compensate loss of LPS to maintain the outer-membrane barrier. The expression patterns of biofilm-associated genes in LPS-modified Col^R^ isolates were not significantly different from their Col^S^ counterparts, whereas this pattern between LPS-deficient Col^R^ isolate and its Col^S^ counterpart was significantly different. According to our findings, it seems that the type of mutation involved in colistin resistance may be decisive on the expression of biofilm-associated genes and biofilm formation capacity among Col^R^ isolates.

Investigation of surface motility showed that the acquisition of colistin resistance was associated with a significant reduction in surface motility in LPS-deficient Col^R^ isolate, but not in LPS-modified Col^R^ isolates. Given that there was a reduction in expression of the type I pilus (*csu* pilus) representative gene, *csuE*, but not the type IV pilus representative gene, *pilT*, after development of colistin resistance in LPS-deficient Col^R^ isolate, the type I pilus (*csu* pilus) appears to be involved in surface motility, but not the type IV pilus. To the best of our knowledge, no studies have compared the surface motility between Col^R^ isolates and Col^S^ parental isolates. Previous studies have also demonstrated no association between surface motility and type IV pili in *A. baumannii* (Harding et al., 2013; Chen et al., 2017). However, other studies have shown that motility is associated with type IV pili in *A. baumannii* (Clemmer et al., 2011; Harding et al., 2013). Besides, our results provide evidence of the association between the surface motility and biofilm formation. Consistently, two previous studies revealed a positive association between the surface motility and biofilm formation capacity (Giles et al., 2015; Chen et al., 2017). Considering the contradictory reports on the association between motility and biofilm formation, and type IV pili, further studies are needed to evaluate surface motility in relation to type IV pili and biofilm formation capacity in *A. baumannii*.

To date, only one report on LPS-deficient Col^R^ *A. baumannii* harboring *lpxD* mutation has been represented, which was isolated following colistin treatment from a patient in South Korea (Park et al., 2009), whereas the remaining reported Col^R^ isolates harbored *pmrAB* mutations (LPS-modified Col^R^ isolates) (Beceiro et al., 2014). An explanation for this condition may be the biological cost of LPS-deficient colistin resistance. Our results indicate that the biological cost of LPS-deficient colistin resistance in terms of growth rate, antibiotic susceptibility, motility, and biofilm formation ability, is higher than the colistin resistance associated with LPS modification. Although the acquisition of colistin resistance mediated by the loss of LPS confers a selective advantage in the presence of colistin treatment, it leads to reduce fitness and biofilm formation capacity and consequently may reduce virulence. A similar explanation was demonstrated by a previous study investigating the effect of colistin resistance-associated mutations on the fitness of *A. baumannii* (Mu et al., 2016). Besides, another possible explanation represented by Boll et al. (2016) may be that some *A. baumannii* strains did not produce LOS-deficient Col^R^ colonies, indicating that some *A. baumannii* clinical isolates could not survive without LOS in the presence of colistin because the expression of PBP1A impeded isolation of LOS-deficient colonies. Collectively, considering to the biological cost of LPS-deficient colistin resistance (in terms of growth rate, antibiotic susceptibility, and biofilm formation ability), and essentiality of LPS for survival of some *A. baumannii* strains, we hypothesize that colistin resistance mediated by mutation in the *pmrAB* gene (LPS modification) will be more likely to arise in patients treated with colistin.

Further researches with a larger sample size appear to be required for better understanding the relationship between the underlying genetic basis of colistin resistance and the accompanying biological features including fitness, growth rate, biofilm formation ability, and other virulence characteristics.

## Ethics Statement

All animal procedures were conducted under protocols approved by the Iranian Ethical Guidelines for the use of animals from the research ethics committee of Tehran University of Medical Sciences.

## Author Contributions

AB and ZF proposed the original idea. All authors developed the protocol and abstracted and analyzed the data. ZF and BT wrote the manuscript and performed the additional experiments as well as the edition of the manuscript.

## Conflict of Interest Statement

The authors declare that the research was conducted in the absence of any commercial or financial relationships that could be construed as a potential conflict of interest. The reviewer EG and handling Editor declared their shared affiliation.

## Footnotes

1 http://www.pasteur.fr/recherche/genopole/PF8/mlst/

2 http://primer3.ut.ee
