# Supplementary material for: Growth Rate and Biofilm Formation Ability of Clinical and Laboratory-Evolved Colistin-Resistant Strains of Acinetobacter baumannii
Source: Front Microbiol. 2018 Feb 12;9:153. doi: 10.3389/fmicb.2018.00153 (PMC5816052; doi:10.3389/fmicb.2018.00153)
Supplement: Supplementary file 1 [file Table_1.DOCX]

**Supplementary table 1.** Oligonucleotides used in multiplex-PCR and qRT-PCR

| **PCR primers** | **Related genes primers** | | | **Primer sequence (5’ to 3’)** | **Amplicon size (bp)** |
| --- | --- | --- | --- | --- | --- |
|  | *abaI** | A1S_0109 | forward | CCACACAACCCTATTTACTCGG | 121 |
|  |  |  | reverse | GGCGGTTTTGAAAAATCTACGG |  |
|  | *ompA** | A1S_2840 | forward | AGCATAAAGAAGCTACACCTGC | 154 |
|  |  |  | reverse | AAAGTCGCCAAGAAACCTTGAT |  |
|  | Type I fimbriae*** | A1S_1510 | forward | GACATTGGTAGCTGCACCAG | 150 |
|  |  |  | reverse | GATGTTGCTGGTCGTACACC |  |
|  | *PilT** | A1S_0897 | forward | AGTGTACCAAACACCAAGTGAC | 284 |
|  |  |  | reverse | TCGGGTAAATCAACTACGCTTG |  |
|  | *bfmR* | A1S_0748 | forward | GGATCTTGTGGTCTTGGATGTC | 384 |
|  |  |  | reverse | GATAAAATACGGCCAGCGTTTG |  |
|  | *bfmS* | A1S_0749 | forward | CACGTATTCGCTTTGGTACAGA | 474 |
|  |  |  | reverse | GGCTATCATCTAAACGGGCAAA |  |
|  | *csuE* | A1S_2213 | forward | TTGGCTTTAGCAAACATGACCT | 564 |
|  |  |  | reverse | TTGCGGGGAAAGTCCATTATTT |  |
|  | *pgaA* | A1S_2162 | forward | GCAAATGAATCCTTCCGATCCT | 670 |
|  |  |  | reverse | GTTTTGAGTCGTTTTTCGCCAT |  |
|  | *bap* | A1S_2696 | forward | GGTACAAACTATGTGCCGGATT | 934 |
|  |  |  | reverse | CTGTATTCACTCCTTGACCAGC |  |
| **qRT-PCR primers** | *bfmR* | A1S_0748 | forward | GTGAAGTTCGCCCACACTAT | 91 |
|  |  |  | reverse | GCACCCATTTCCAAACCAAG |  |
|  | *bfmS* | A1S_0749 | forward | GGGCAAAGGCTTCAAATACAC | 108 |
|  |  |  | reverse | GGATTACGGGCGGTATTCATAA |  |
|  | *csuE* | A1S_2213 | forward | ACCTTTCTACATACGGCTTCC | 104 |
|  |  |  | reverse | CAACTGCGGGTACAGAATAGA |  |
|  | *pgaA* | A1S_2162 | forward | TTGATCCAGATGATTAGCGTAGG | 99 |
|  |  |  | reverse | AGTCAGGCTAAGGGTGTAGATA |  |
|  | *bap* | A1S_2696 | forward | AATGCACCGGTACTTGATCC | 250 |
|  |  |  | reverse | TATTGC CTGCAGGGTCAGTT |  |
|  | 16SrRNA | - | forward | AAAGTTGGTATTCGCAACGG | 117 |
|  |  |  | reverse | ACCTTTAACCCGCTTTTGCT |  |

**abaI* , *PilT*, Type I fimbriae (A1S_1510), and *ompA* primers were used for both reactions PCR and qRT-PCR
