# Supplementary material for: Growth Rate and Biofilm Formation Ability of Clinical and Laboratory-Evolved Colistin-Resistant Strains of Acinetobacter baumannii
Source: Front Microbiol. 2018 Feb 12;9:153. doi: 10.3389/fmicb.2018.00153 (PMC5816052; doi:10.3389/fmicb.2018.00153)
Supplement: Supplementary file 2 [file Table_2.docx]

**Supplementary Table 2.** Minimum inhibitory concentrations (MICs) of *A. baumannii* strains for 21 antibiotics

| **Antimicrobial agents*** | **MIC of isolates to antimicrobial agents (µg/ml)** | | | | | | | | | | | |
| --- | --- | --- | --- | --- | --- | --- | --- | --- | --- | --- | --- | --- |
|  | **Ab12** | **Ab12R** | **Fold** | **Ab99** | **Ab99R** | **Fold** | **Ab1** | **Ab2** | **Fold** | **Ab321** | **Ab328** | **Fold** |
| **TIC** | 128 | 32 | 4 | 64 | 64 | 1 | 128 | 128 | 1 | 256 | 256 | 1 |
| **PIP** | 256 | 128 | 2 | 256 | 256 | 1 | 64 | 64 | 1 | 128 | 128 | 1 |
| **TZP** | 128 | 64 | 2 | 128 | 128 | 1 | 64 | 64 | 1 | 128 | 128 | 1 |
| **SAM** | 16 | 8 | 2 | 16 | 8 | 2 | 4 | 4 | 1 | 32 | 32 | 1 |
| **CRO** | 64 | 64 | 1 | 32 | 32 | 1 | 64 | 64 | 1 | 128 | 128 | 1 |
| **CTX** | 64 | 32 | 2 | 32 | 32 | 1 | 64 | 64 | 1 | 64 | 64 | 1 |
| **CAZ** | 64 | 32 | 2 | 32 | 32 | 1 | 64 | 64 | 1 | 16 | 16 | 1 |
| **FEP** | 64 | 16 | 4 | 64 | 64 | 1 | 32 | 32 | 1 | 32 | 32 | 1 |
| **IMP** | 32 | 4 | 8 | 16 | 16 | 1 | 64 | 64 | 1 | 32 | 32 | 1 |
| **MEM** | 16 | 4 | 4 | 32 | 32 | 1 | 16 | 16 | 1 | 16 | 16 | 1 |
| **TOB** | 128 | 32 | 4 | 64 | 64 | 1 | 64 | 64 | 1 | 32 | 32 | 1 |
| **GEN** | 64 | 16 | 4 | 32 | 32 | 1 | 128 | 128 | 1 | 128 | 128 | 1 |
| **AMK** | 32 | 4 | 8 | 8 | 4 | 2 | 16 | 16 | 1 | 32 | 16 | 2 |
| **TET** | 16 | 16 | 1 | 16 | 16 | 1 | 16 | 16 | 1 | 32 | 32 | 1 |
| **MIN** | 16 | 16 | 1 | 8 | 8 | 1 | 16 | 16 | 1 | 16 | 8 | 2 |
| **DOX** | 8 | 8 | 1 | 8 | 8 | 1 | 16 | 16 | 1 | 8 | 8 | 1 |
| **CIP** | 32 | 64 | 2 | 16 | 16 | 1 | 32 | 32 | 1 | 32 | 32 | 1 |
| **RIF** | 8 | 0.5 | 16 | 2 | 2 | 1 | 8 | 8 | 1 | 8 | 4 | 2 |
| **TGC** | 2 | 8 | 4 | 2 | 2 | 1 | 2 | 2 | 1 | 4 | 4 | 1 |
| **AZM** | 256 | 2 | 128 | 256 | 256 | 1 | 256 | 256 | 1 | 128 | 128 | 1 |
| **VAN** | 256 | 1 | 256 | 128 | 128 | 1 | 128 | 128 | 1 | 256 | 256 | 1 |

*Abbreviations: AMK, amikacin; AZM, azithromycin; CAZ, ceftazidime; CTX, cefotaxime; CRO, ceftriaxone; CIP, ciprofloxacin; FEP, cefepime; GEN, gentamicin; IPM, imipenem; MEM, meropenem; MIN, minocycline; PIP, piperacillin; RIF, rifampin; SAM, ampicillin-sulbactam; SXT, trimethoprim/sulfamethoxazole; TET, tetracycline; TIC, ticarcillin; TGC, tigecycline; TOB, tobramycin; TZP, piperacillin-tazobactam; VAN, vancomycin.
