# Supplementary material for: Growth Rate and Biofilm Formation Ability of Clinical and Laboratory-Evolved Colistin-Resistant Strains of Acinetobacter baumannii
Source: Front Microbiol. 2018 Feb 12;9:153. doi: 10.3389/fmicb.2018.00153 (PMC5816052; doi:10.3389/fmicb.2018.00153)
Supplement: Supplementary file 3 [file Table_3.DOCX]

**Supplementary table 3.** The biofilm formation capacity among Col^R^ and Col^S^ isolates in both conditions *in vitro* and *in vivo*

|  | ***In vitro* biofilm formation (mean ± standard devision)** | | | | | | ***In vivo* biofilm formation (mean ± standard devision)** | | | | | | **Motility assay** | |
| --- | --- | --- | --- | --- | --- | --- | --- | --- | --- | --- | --- | --- | --- | --- |
| **Isolates^a^** | **Microplate assay** | | | **Catheter-associated biofilm** | | |  |  |  |  |  |  |  |  |
|  | **CV^b^ assay** | | | **Colony count (log_10_)** | | | **CV assay** | | | **XTT^c^ assay** | | | colony diameter (cm)± SD | **P. value** |
|  | **^d^OD570** | **^e^SD** | **P value** | **Count** | **SD** | **P value** | **OD570** | **SD** | **P value** | **OD492** | **SD** | **P value** |  |  |
| **Ab12** | 0.164 | 0.026 | 0.021 | 6.7 | 0.015 | 0.001 | 0.324 | 0.024 | 0.0001 | 0.460 | 0.034 | 0.0001 | 5.3±1.1 | 0.035 |
| **Ab12R** | 0.069 | 0.005 |  | 5.3 | 0.1 |  | 0.086 | 0.013 |  | 0.082 | 0.009 |  | 3.1±0.7 |  |
| **Ab99** | 0.229 | 0.0015 | 0.627 | 7.76 | 0.057 | 0.26 | 0.261 | 0.009 | 0.713 | 0.477 | 0.045 | 0.71 | 6.1±1.1 | 0.082 |
| **Ab99R** | 0.223 | 0.015 |  | 7.71 | 0.045 |  | 0.270 | 0.039 |  | 0.462 | 0.044 |  | 5.1±0.9 |  |
| **Ab1** | 0.288 | 0.013 | 0.075 | 8.56 | 0.055 | 0.052 | 0.417 | 0.014 | 0.225 | 0.381 | 0.003 | 0.116 | 5.4±0.8 | 0.091 |
| **Ab2** | 0.242 | 0.030 |  | 8.46 | 0.025 |  | 0.375 | 0.042 |  | 0.361 | 0.017 |  | 5.6±0.7 |  |
| **Ab321** | 0.304 | 0.030 | 0.78 | 8.28 | 0.59 | 0.392 | 0.453 | 0.017 | 0.282 | 0.420 | 0.007 | 0.985 | 5.7± 0.9 | 0.083 |
| **Ab328** | 0.298 | 0.021 |  | 8.65 | 0.03 |  | 0.432 | 0.023 |  | 0.421 | 0.028 |  | 5.2 ± 0.8 |  |

^a^Colistin susceptible (Col^S^) isolates: Ab12, Ab99, Ab1 and Ab321; Colistin resistant (Col^R^) isolates: Ab12R, Ab99R, Ab2 and Ab328. ^b^CV, crystal violet; ^c^XTT, 2,3-Bis-(2-Methoxy-4-Nitro-5-Sulfophenyl)-2H-Tetrazolium-5-Carboxanilide; ^d^OD, optical density; ^e^SD, standard deviation.
